# Bacteremia in critical care units at Bugando Medical Centre, Mwanza, Tanzania: the role of colonization and contaminated cots and mothers’ hands in cross-transmission of multidrug resistant Gram-negative bacteria

**DOI:** 10.1186/s13756-020-00721-w

**Published:** 2020-05-06

**Authors:** Vitus Silago, Dory Kovacs, Delfina R. Msanga, Jeremiah Seni, Louise Matthews, Katarina Oravcová, Ruth N. Zadoks, Athumani M. Lupindu, Abubakar S. Hoza, Stephen E. Mshana

**Affiliations:** 1 Department of Microbiology and Immunology, Weill Bugando School of Medicine, Catholic University of Health and Allied Sciences, P. O. Box 1464, Bugando, Mwanza, Tanzania; 2 Department of Veterinary Microbiology, Parasitology and Biotechnology, College of Veterinary Medicine and Biomedical Sciences, Sokoine University of Agriculture, P. O. Box 3000, Morogoro, Tanzania; 3 Institute of Biodiversity, Animal Health and Comparative Medicine, University of Glasgow, Glasgow, UK; 4 Department of Pediatrics and Child Health, Weill Bugando School of Medicine, Catholic University of Health and Allied Sciences, P. O. Box 1464, Bugando, Mwanza, Tanzania; 5 Sydney School of Veterinary Science, University of Sydney, Sydney, Australia; 6 Department of Veterinary Medicine and Public Health, College of Veterinary Medicine and Biomedical Sciences, Sokoine University of Agriculture, P. O. Box 3000, Morogoro, Tanzania

**Keywords:** Antimicrobial resistance, Hand hygiene, Hospital surfaces contamination, Multidrug resistant bacteria, Bacteremia

## Abstract

**Background:**

Multidrug resistance (MDR) is a major clinical problem in tertiary hospitals in Tanzania and jeopardizes the life of neonates in critical care units (CCUs). To better understand methods for prevention of MDR infections, this study aimed to determine, among other factors, the role of MDR-Gram-negative bacteria (GNB) contaminating neonatal cots and hands of mothers as possible role in transmission of bacteremia at Bugando Medical Centre (BMC), Mwanza, Tanzania.

**Methods:**

This cross-sectional, hospital-based study was conducted among neonates and their mothers in a neonatal intensive care unit and a neonatology unit at BMC from December 2018 to April 2019. Blood specimens (*n* = 200) were sub-cultured on 5% sheep blood agar (SBA) and MacConkey agar (MCA) plates. Other specimens (200 neonatal rectal swabs, 200 maternal hand swabs and 200 neonatal cot swabs) were directly inoculated on MCA plates supplemented with 2 μg/ml cefotaxime (MCA-C) for screening of GNB resistant to third generation cephalosporins, r-3GCs. Conventional biochemical tests, Kirby-Bauer technique and resistance to cefoxitin 30 μg were used for identification of bacteria, antibiotic susceptibility testing and detection of MDR-GNB and screening of potential Amp-C beta lactamase producing GNB, respectively.

**Results:**

The prevalence of culture confirmed bacteremia was 34.5% of which 85.5% were GNB. Fifty-five (93.2%) of GNB isolated from neonatal blood specimens were r-3GCs. On the other hand; 43% of neonates were colonized with GNB r-3GCs, 32% of cots were contaminated with GNB r-3GCs and 18.5% of hands of neonates’ mothers were contaminated with GNB r-3GCs. The prevalences of MDR-GNB isolated from blood culture and GNB r-3GCs isolated from neonatal colonization, cots and mothers’ hands were 96.6, 100, 100 and 94.6%, respectively. Significantly, cyanosis (OR[95%CI]: 3.13[1.51–6.51], *p* = 0.002), jaundice (OR[95%CI]: 2.10[1.07–4.14], *p* = 0.031), number of invasive devices (OR[95%CI]: 2.52[1.08–5.85], p = 0.031) and contaminated cot (OR[95%CI]: 2.39[1.26–4.55], *p* = 0.008) were associated with bacteremia due to GNB. Use of tap water only (OR[95%CI]: 2.12[0.88–5.09], *p* = 0.040) was protective for bacteremia due to GNB.

**Conclusion:**

High prevalence of MDR-GNB bacteremia and intestinal colonization, and MDR-GNB contaminating cots and mothers’ hands was observed. Improved cots decontamination strategies is crucial to limit the spread of MDR-GNB. Further, clinical presentations and water use should be considered in administration of empirical therapy whilst awaiting culture results.

## Background

Multidrug resistance (MDR) is defined as acquired resistance to at least one agent in three or more antimicrobial classes [1–3]. MDR is a growing global concern which is estimated to cause 10 million deaths and cost US$100 trillion annually by 2050 [4, 5]. Improper use of antibiotics in human and veterinary medicine, counterfeit antibiotics and non-compliant use of rationally prescribed antibiotics are among factors driving the emergence and spread of MDR bacteria [6]. High antibiotic pressure (empirically prescribed and administered) in the critical care units (intensive care units and neonatology units) results in the selection and emergence of MDR bacteria [7, 8]. The spread of MDR bacteria in healthcare settings presents a challenge, as treating infected patients becomes increasingly difficult with poorer outcomes [6]. MDR Gram-negative bacteria (MDR-GNB) such as beta-lactamase (extended spectrum beta-lactamase (ESBL), Amp-C beta-lactamase and carbapenemases) producing Enterobacteriaceae, *Acinetobacter baumannii* and *Pseudomonas aeruginosa* are frequently reported, causing infections in critical care units globally [9–12]. These organisms are responsible for bloodstream infections (BSIs), urinary tract infections (UTIs), pneumonia, and skin and soft tissue infections, resulting in high morbidity and mortality [13].

In critical care units, infections due to MDR-GNB bacteria may be acquired endogenously or exogenously [14]. Endogenous acquisition occurs from the patient’s own body flora colonizing a certain body surface, for example, MDR-GNB colonizing patient’s gastrointestinal tract (such as MDR-*E. coli*) may cause an extra-intestinal infection (e.g., BSI and UTI) which may even result to mortality from treatment failure [14, 15]. Exogenous acquisition occurs due to contact with other people e.g., healthcare workers (HCWs), patients or care givers (CGs) i.e., mothers; and/or contaminated surfaces, such as ventilators, beds, side tables and infusion stands, and water sources [14, 16]. Contaminated patients’ environments with MDR-GNB increases the risk of exogenous acquire of healthcare associate infections (HCAIs) from MDR-GNB which are mostly cross-transmitted by contaminated hands of HCWs and CGs [17]. Hands of HCWs and CGs become contaminated when touching contaminated surfaces and even colonized patients during provision of medical care [18].

In Mwanza, Tanzania, 10.5 to 49% of bacteremia cases due to GNB are caused by MDR-GNB with mortality rate ranging from 34.4 to 52% as compared to mortality with none MDR-GNB which ranging from 16.2 to 25% [11, 19, 20]. Rectal colonization of neonates with extended-spectrum beta-lactamase producing Enterobacteriaceae (ESBL-PE) is common (25.4 to 54.6%) [19, 20], as is contamination of the hospital’s inanimate surfaces (33.5%) [21]. In Tanzania, it is common that, mothers play an important role in feeding and caring for hospitalized neonates. To date, their role in infection transmission or prevention has not been considered. To reduce the incidence and improve the management of MDR-GNB cases in the neonatal ICU and neonatology unit at BMC, we explored exogenous and endogenous risk factors for neonatal MDR-GNB sepsis, including potential exogenous exposures in the household of origin, in the hospital or from mothers, and endogenous exposure (neonatal carriage). Results can be used to inform case management, and to target infection prevention and control measures to reduce case incidence.

## Methods

### The aim, design and setting of the study

A cross-sectional hospital-based study was conducted between December 2018 and July 2019 aimed to determine, among other factors, the role of MDR-Gram-negative bacteria (GNB) contaminating neonatal cots and hands of mothers as possible role in transmission of bacteremia among neonates admitted to the neonatal ICU (NICU) and neonatology unit at Bugando Medical Centre (BMC), Mwanza, Tanzania. BMC is a tertiary, teaching, consultancy and zonal referral hospital with an estimated 1000-bed capacity, serving Lake Zone regions (Mwanza, Simiyu, Kagera, Shinyanga, Musoma, Tabora, Geita and Kigoma) and a catchment population of 13 million people (https://www.bugandomedicalcentre.go.tz/index.php). The NICU was equipped with 15 neonatal cots (with no walking space between cots), 15 trained nurses and 2 pediatricians. In the neonatology unit, there were 36 cots about 0.5 m apart, 11 trained nurses and 4 pediatricians. When operating at or above capacity, two neonates may share a cot (in both units). In both units, the cots are irregularly disinfected before new occupancy by using 1:50 Dettol in water.

### Sample size calculation and selection criteria

A minimum sample size for this study was 144 participants, which was calculated using Kish Leslie formula of 1965 [22], using an MDR-GNB prevalence of 10.5% [20]. Neonates admitted to NICU and neonatology unit with signs and symptoms of infections as previous reported by “WHO Young infants Study group” [23] and their mothers were enrolled in this study. Neonates with signs and symptoms of infection but either missing socio-demographic information or a complete set of specimens were excluded from the final analysis (*n* = 15). Participants (neonates and mothers) moving between the neonatal ICU and neonatology units were not re-enrolled.

### Data and specimen collection

Structured questionnaires were used to obtain socio-demographic and clinical information from study participants after the mother or guardian consented to participation. Neonatal blood samples, neonatal rectal swabs, cot swabs and maternal hand swabs were collected. About 1 ml of venous blood was collected into an in-house made tryptone soy broth (TSB, 10 ml) by paediatrician; rectal swabs were collected by a trained medical doctor; and bed swabs (in every new occupancy) and mothers’ hand swabs specimens were collected. All swab samples were collected using sterile cotton swabs pre-moistened in sterile 0.85% physiological saline. All swab specimens were transported to the laboratory in Amies transport media (Amies, UK). In total, 800 specimens (200 blood, 200 rectal swabs, 200 bed swabs and 200 mothers’ hands swabs) were collected. All specimens were sent to the microbiology laboratory of the Catholic University of Health and Allied Sciences for isolation, identification, antibiotic susceptibility testing and detection of MDR-GNB following in-house standard operating procedures and international guidelines such as Clinical and Laboratory Standard Institute (CLSI, 2018) [24].

### Definitions

In this study, GNB isolated from blood with resistance to ceftriaxone and/or ceftazidime and GNB isolated from rectal, bed and hand swabs grown on MacConkey agar plates supplemented with 2 μg/ml cefotaxime (MCA-C) were considered resistant to third generation cephalosporins (r-3GCs) [11]. All GNB isolated from neonates’ blood, rectal, cots and mothers’ hands swab specimens showing resistance to at least one antibiotic agent in three different classes of antibiotics i.e., penicillins: ampicillin (AMP), amoxicillin/clavulanate (AMC), piperacillin/tazobactam (TZP); third generation cephalosporins (3GCs): ceftriaxone (CRO), ceftazidime (CAZ) and/or isolated on MCA-C; carbapenems: meropenem (MEM); trimethoprim-sulfamethoxazole (SXT); aminoglycosides: gentamicin (CN), amikacin (AK); fluoroquinolones: ciprofloxacin (CIP); tetracyclines: tetracycline (TET); and /or polymyxins: colistin (CT), were termed as MDR-GNB as previously reported [1, 2]. In this paper, isolates exhibiting intermediate activities against antibiotics were also termed as resistant.

#### Laboratory procedures

##### Bacterial isolation, identification and antibiotic susceptibility testing

***Clinical specimens (blood):*** Blood specimens in TSB bottles were incubated aerobically at 37 °C for 18–24 h upon receipt in the laboratory, and before being inoculated onto in-house prepared 5% sheep blood agar (SBA) and MacConkey agar (MCA) plates (Oxoid, UK). SBA and MCA plates were incubated aerobically at 37 °C for 18–24 h. However isolation of Gram positive bacteria was not the objective of this study, we purposely isolated and identified them and their antibiotic susceptibility testing were performed to guide rational antibiotic therapy for proper patients’ management only.

Isolated bacteria were identified by in-house prepared conventional biochemical identification tests including sugars fermentation, CO_2_ gas production and sulfur production by triple sugar iron (TSI) test; sulfur production, indole production and motility by sulfur-indole-motility (SIM) test, urease production by urease test; utilization of citrate as the sole source of energy by Simmons’ citrate test; and oxidase production by oxidase test strips as reported previously [25]. Kirby-Bauer disc diffusion method was used for antibiotics susceptibility testing (AST) on MHA plates [26]. Briefly, bacterial suspensions equivalent to 0.5 McFarland turbidity standard solution were prepared from a MacConkey subculture (arising from a cultured clinical specimen and one isolated colony from cefotaxime-supplemented MacConkey agar) into sterile 0.85% physiological saline and then swabbed on entire plates of MHA (Oxoid, UK). Ampicillin (AMP) 10 μg, trimethoprim-sulfamethoxazole (SXT) 25 μg, amikacin (AK) 30 μg, tetracycline (TE) 30 μg, piperacillin-tazobactam (TZP) 110 μg, gentamicin (CN) 10 μg, ciprofloxacin (CIP) 5 μg, amoxicillin-clavulanic acid (AMC) 30 μg, ceftriaxone (CRO) 30 μg, ceftazidime (CAZ) 30 μg, meropenem (MEM) 10 μg and colistin sulfate (CT) 10 μg antibiotic discs (Oxoid, UK) were seeded onto inoculated MHA plates within 15 min. Interpretation of zones of inhibitions was done according to CLSI, 2018 [27]. Cefoxitin (FOX) 30 μg discs were also included in AST purposely for screening of potential Amp-C beta lactamase producing GNB. Isolates exhibiting zone diameters ≤18 mm were considered potential Amp-C beta lactamase producers as reported previous [28, 29]. Zone diameters for CT were interpreted as previous reported by Galani et al. 2008 [30].

***Colonization and contamination specimens (rectal, cot and hand swabs):*** Immediately upon receipt of swab specimens in the laboratory, these were inoculated on MCA-C (Medochemie Ltd., Cyprus) for isolation of MDR-GNB. Plates were incubated aerobically at 37 °C for 18–24 h. Conventional biochemical identification tests were used for characterisation of isolates to species levels as described earlier. For AST, the antimicrobial panels and concentrations were as described above, but beta-lactam antibiotic discs were excluded as isolation of resistant GNB involved the use of cefotaxime (beta-lactam) 2 μg/ml supplemented MCA plates. CLSI (2018) [27] and Galani et al. 2008 [30] guidelines were used for interpretation of zones of inhibitions.

### Statistical analysis

STATA software version 13.0 was used for data analysis. Continuous data were presented as median (interquartile range) whereby categorical data were presented as percentages and fractions. Logistic regression and a stepwise backwards model selection analysis was used to determine risk factors and clinical symptoms for neonatal bacteremia in critical care units. A *p* value less than 0.05 at 95% confidence interval was considered statistical significant.

## Results

### Socio-demographic and clinical characteristics of neonates admitted in neonatal ICU and neonatology unit at BMC

Two-hundred neonates with median age (interquartile range) of 1 (1–2) days were enrolled during this study period, including 52.5% males and 47.5% females. Just over half of the neonates (58%) were enrolled from the neonatology unit. The median duration (interquartile range) of a hospital stay was 7 (1–22.5) days. The majority of neonates (73%), were enrolled after > 48 h of admission and 87.5% were on antibiotic treatment at the time of clinical sampling and 24.5 and 84% had fever and invasive devices during enrolment, respectively. In-unit mortality was 9% in either unit (Table 1).

**Table 1:** Socio-demographic and clinical characteristics of neonates admitted in neonatal ICU and neonatology unit at BMC

Characteristics		Frequency (n)	Percentage (%)
Sex (*N* = 200)	Females	95	47.5
	Males	105	52.5
Unit (N = 200)	Neonatology unit	116	58
	Neonatal ICU	84	42
Keeping livestock at home (N = 200)	Yes	37	18.5
	No	163	81.5
Keeping pet* at home (N = 200)	Yes	74	37
	No	126	63
Water sources (N = 200)	Open sources	14	7
	Tap water	175	87.5
	Both	11	5.5
Drinking water treatment (boiling) (N = 200)	Yes	123	61.5
	No	77	38.5
Fever during sampling (N = 200)	Yes	35	17.5
	No	165	82.5
Type of fever (*N* = 35)	Hypothermia	16	45.7
	Hyperthermia	19	54.3
Heart rate (*N* = 192)	Normal	145	75.5
	Abnormal	47	24.5
Breathing/respiration rate (*N* = 191)	Normal	145	75.9
	Abnormal	46	24.1
Oxygen saturation (N = 192)	Normal	142	73.9
	Abnormal	50	26.1
Prematurity status (N = 200)	Yes	144	72.0
	No	56	28.0
Length of hospital stay at enrollment (*N* = 200)	< 48 h	143	71.5
	> 48 h	57	28.5
On antibiotics at the time of clinical sampling (N = 200)	Yes	175	87.5
	No	25	12.5
Type of antibiotic (*N* = 175)	Ceftriaxone	3	1.7
	Gentamicin	166	94.9
	Ampicillin/ampiclox	171	97.7
Presence of invasive device at sampling (N = 200)	Yes	166	83.0
	No	34	17.0
Type of invasive device (*N* = 166)	Urinary catheter (UC)	5	3.0
	Nasogastric tube (NT)	125	75.3
	Intravenous line (IV line)	161	96.9
	IV line + NT	117	70.5
	IV line + NT + UC	5	3.0
Convulsion (N = 192)	Yes	7	3.6
	No	185	96.4
Paleness (N = 200)	Yes	22	11.0
	No	178	89.0
Meconium stained (N = 200)	Yes	33	16.5
	No	167	83.5
Resuscitation (N = 192)	Yes	128	66.7
	No	64	33.3
Poor feeding (N = 192)	No	59	30.7
	Yes	133	69.3
Jaundice (N = 200)	Yes	64	32.0
	No	136	68.0
Cyanosis (N = 200)	Yes	53	26.5
	No	147	73.5
Nasal flaring (N = 200)	Yes	111	55.5
	No	89	44.5
Chest indrawing (N = 200)	Yes	78	39.0
	No	122	61.0
Discharging umbilical cord (N = 200)	Yes	60	30.0
	No	140	70.0
Outcomes (N = 200)	Death	18	9.0
	Discharge	182	91.0

Notes: *IQR* interquartile range; Median age (IQR) in days: 1 (1–2) days; Median days (IQR) of hospital stay: 7 (1–22.5) days and *pet = dog and/or cat

### Culture results; blood, rectal, neonatal cots and mothers’ hands specimens

The prevalence of culture confirmed bacteremia was 34.5% of which 85.5% were GNB. About 93.2% of the GNB isolated from positive blood cultures were r-3GCs. The prevalence of GNB r-3GCs (grown MCA-C) colonizing neonates, contaminating neonates’ cots and mothers’ hands was 43, 32 and 18.5%, respectively. *K. pneumoniae*, *Acinetobacter* spp., *E. coli* and *C. freundii* were frequently isolated from neonates’ blood and rectal swab specimens suggesting that rectal colonization may be the source of bacteremia. On the other hand, *K. pneumoniae*, *Acinetobacter* spp. and *E. aerogenes* were frequently isolated from neonates’ cots and mothers’ hands suggesting possibilities of mothers’ hands get contaminated when touching contaminated neonates’ cots. The incidence of potential Amp-C beta lactamase producers was higher among isolates contaminating neonates’ cots and mothers’ hands, respectively (Table 2).

**Table 2:** Culture results: blood, rectal swab, cot swab and mothers’ hands swab specimens

Variables		Blood culture		Rectal culture		Cots culture		CGs’ hands culture	
		n	%	n	%	n	%	n	%
Culture results	Positive	69	34.5	86	43	64	32	37	18.5
	Negative	131	65.5	114	57	136	68	163	81.5
Classification of positive blood culture	Gram-positive	10	14.5	NA	NA	NA	NA	NA	NA
	Gram-negative	59	85.5	NA	NA	NA	NA	NA	NA
Genus and species of isolated bacteria^#^	*K. pneumoniae*	28	47.5	49	45.4	18	28.1	17	45.9
	*Acinetobacter* spp	19	32.2	23	21.3	35	54.7	8	21.6
	*E. coli*	5	8.5	14	12.9	1	1.6	2	5.4
	*C. freundii*	3	5.1	10	9.3	3	4.7	1	2.7
	*E. aerogenes*	1	1.7	4	3.7	6	9.4	5	13.5
	Others*	3	5.1	8	7.4	1	1.6	4	10.8
Resistant to 3GCs (blood culture only)	Positive	55	93.2	NA	NA	NA	NA	NA	NA
	Negative	4	6.8	NA	NA	NA	NA	NA	NA
Amp-C beta lactamase (FOX≤18 mm)	Positive	23	38.9	50	46.3	48	75	22	59.5
	Negative	36	61.1	58	53.7	16	25	15	40.5
Genus and species of potential Amp-C beta lactamase producers	*Acinetobacter* spp	13	56.5	22	44	34	70.8	8	36.4
	*C. freundii*	3	13.0	7	14	3	6.3	1	4.5
	*K. pneumoniae*	2	8.7	12	24	4	8.3	5	22.7
	*E. coli*	2	8.7	2	4	1	2.1	1	4.5
	Others**	3	12.9	7	14	6	12.5	7	31.8

^#^Blood culture: GNB only

*Blood culture: *E. cloacae* (*n* = 1), *Salmonella* spp. (*n* = 1) and unidentified GNB (n = 1)

*Rectal swabs: *E. cloacae* (*n* = 2), *Shigella* spp. (*n* = 2), *P. aeruginosa* (*n* = 1), *Salmonella* spp. (*n* = 1), *K. oxytoca* (*n* = 1) and *P. agglomerans* (*n* = 1)

*Neonatal cot swabs: *A. hydrophila* (*n* = 1)

*Mothers’ hands swabs: *E. cloacae* (*n* = 3), *K. oxytoca* (*n* = 1)

**Blood culture: *E. aerogenes* (*n* = 1), *Salmonella* spp. (*n* = 1) and unidentified GNB (n = 1)

**Rectal swabs: *E. aerogenes* (*n* = 3), *E. cloacae* (*n* = 2), *P. aeruginosa* (n = 1) and *Salmonella* spp. (n = 1)

**Neonatal cot swabs: *E. aerogenes* (*n* = 6)

**Mothers’ hands swabs: *E. aerogenes* (*n* = 4), *E. cloacae* (*n* = 2) and *K. oxytoca* (n = 1)

### Percentage resistance of GNB isolated from blood culture and GNB r-3GCs isolated from rectal, cots and hands swabs specimens and respective magnitude of MDR-GNB

**Fig. 1:**
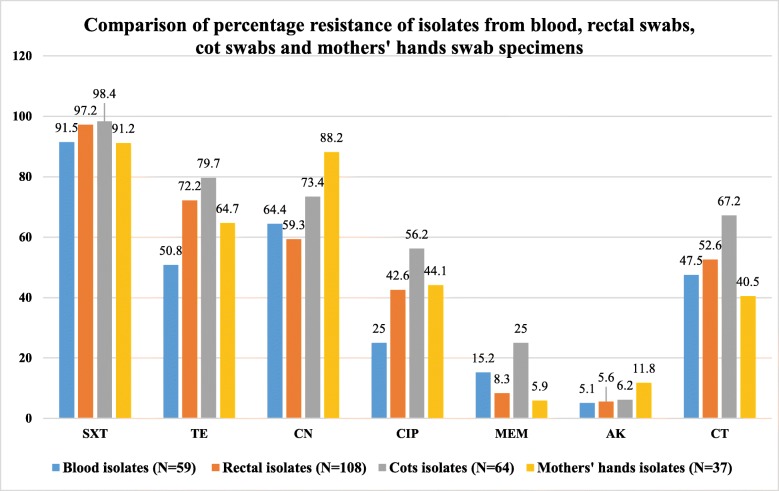
Comparison of percentage resistance of isolates from blood, rectal swab, cot swabs and mothers’ hands swab specimens against antibiotic agents tested in common

**Fig. 2:**
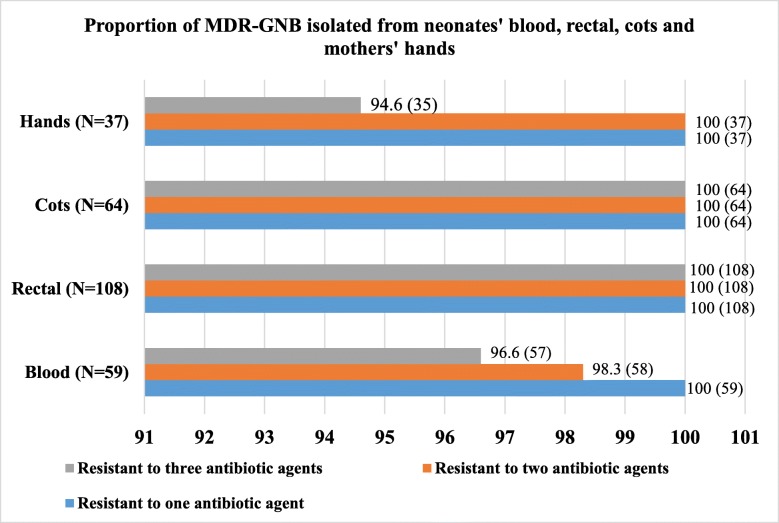
Proportion of MDR-GNB isolated from neonates’ blood, rectal, cots and mothers’ hands. The number of isolates in indicated in brackets

More than 90% of GNB isolated from blood exhibited resistance to AMP, SXT, AMC and CRO. Isolates colonizing neonates and contaminating their cots had similar frequencies of antibiotics resistance. Both exhibited more than 95 and 70% resistance to STX and TE, respectively. GNB contaminating mothers’ hands were highly resistant to SXT (> 90%) and CN (> 85%). GNB contaminating cots were more resistant to CT (67.2%) compared to GNB isolated from blood (47.5%), rectal swabs (52.6%) and mothers’ hand swabs (40.5%). Comparison of common antibiotic agents tested against all isolates is reported below in Fig. 1. Over 90% of GNB isolated from blood, rectal swabs, neonates’ cots and hands of neonates’ mothers were MDR-GNB (resistant to one or more antibiotic agents in three different classes of antibiotics), Fig. 2.

#### Factors associated with bacteremia in critical care units

On multivariate regression analysis, cyanosis (OR[95%CI]: 3.13[1.51–6.51], *p* = 0.002), jaundice (OR[95%CI]: 2.10[1.07–4.14], *p* = 0.031), number of invasive devices (OR[95%CI]: 2.52[1.08–5.85], *p* = 0.031), maternal fever during pregnancy (OR[95%CI]: 2.17[1.17-4.05], *p* = 0.014) and contaminated cot with MDR-GNB (OR[95%CI]: 2.39[1.26–4.55], *p* = 0.008) found to be significantly associated with bacteremia due to GNB. The use of tap water only (OR[95%CI]: 2.12[0.88–5.09], *p* = 0.040) was protective for bacteremia due to GNB (Table 3). In addition, neonates colonized with MDR-GNB, their cots were also significantly contaminated with MDR-GNB (OR[95%CI]: 2.43[1.33–4.47], *p* = 0.004).

**Table 3:** Factors associated with neonatal bacteremia in critical care units

Variable	All participants	Bacteremia by GNB	Univariate	Multivariate	
		***N*** = 59 (%)	***P*** value	OR [95%CI]	***P*** value
**Sex (*N*= 200)**					
Females	95	28 (29.5)	0.994	1.01 [0.54-1.88]	0.972
Males	105	31 (29.5)			
**Unit (*N*= 200)**					
Neonatology unit	116	33 (28.5)	0.702	1.13 [0.60-2.11]	0.700
Neonatal ICU	84	26 (30.9)			
**Length of hospital stay at enrolment (*N*= 200)**					
<48 hours	143	37 (25.9)	0.075	1.80 [0.94-3.46]	0.077
>48 hours	57	22 (39.3)			
**Livestock keeping at home (*N*= 200)**					
No	163	48 (29.5)	0.973	1.02 [0.41-2.53]	0.968
Yes	37	11 (29.7)			
Keeping pet* at home (*N*= 200)					
No	126	37 (29.4)	0.956	0.98 [0.47-2.07]	0.973
Yes	74	22 (29.7)			
**Source of water (*N*= 200)**					
Both	11	7 (63.6)	0.038	2.12 [0.88-5.09]	0.040
Open sources	14	4 (28.6)			
Tap water	175	48 (27.4)			
**Body temperature (*N*= 200)**					
Normal	165	44 (26.7)	0.056	1.55 [0.95-2.55]	0.081
Hypo/hyperthermia	35	15 (42.9)			
**On antibiotic during sampling (*N*= 200)**					
No	25	10 (40.0)	0.218	0.50 [0.18-1.37]	0.180
Yes	175	49 (28.9)			
**Presence of invasive device (*N*= 200)**					
No	34	10 (29.4)	0.990	0.94 [0.36-2.49]	0.909
Yes	166	49 (29.5)			
**Number of invasive devices (*N*= 200)**					
≤1	122	42 (34.4)	0.058	2.52 [1.08-5.85]	0.031
≥2	78	17 (21.8)			
**Prematurity status (*N*= 200)**					
No	56	21 (37.5)	0.124	0.59 [0.30-1.17]	0.135
Yes	144	38 (26.4)			
**Resuscitation (*N*= 192)**					
No	64	17 (26.6)	0.734	0.78 [0.35-1.79]	0.569
Yes	128	37 (28.9)			
**Poor feeding (*N*= 192)**					
No	59	14 (23.7)	0.368	1.07 [0.41-2.85]	0.885
Yes	133	40 (30.1)			
**Convulsion (*N*= 192)**					
No	185	50 (27.0)	0.082	4.60 [0.88-23.78]	0.069
Yes	7	4 (57.1)			
**Paleness (*N*= 200)**					
No	178	52 (29.2)	0.801	1.29 [0.48-3.53]	0.607
Yes	22	7 (31.8)			
**Jaundice (*N*= 200)**					
Negative	136	34 (25.0)	0.043	2.10 [1.07-4.14]	0.031
Positive	64	25 (39.1)			
**Cyanosis (*N*= 200)**					
Negative	147	34 (23.1)	<0.001	3.13 [1.51-6.51]	0.002
Positive	53	25 (47.2)			
**Nasal flaring (*N*= 200)**					
Negative	89	24 (26.9)	0.482	0.86 [0.42-1.76]	0.688
Positive	111	35 (31.5)			
**Chest indrawing (*N*= 200)**					
Negative	122	31 (25.4)	0.114	1.83 [0.94-3.57]	0.076
Positive	78	28 (35.9)			
**Discharging umbilicus (*N*= 200)**					
Negative	140	40 (28.6)	0.660	1.55 [0.755-3.18]	0.232
Positive	60	19 (31.7)			
**Rectal colonization (*N*= 200)**					
Negative	114	28 (24.6)	0.079	1.82 [0.93-3.57]	0.079
Positive	86	31 (36.1)			
**Cot contamination (*N*= 200)**					
Negative	136	32 (23.5)	0.008	2.39 [1.26-4.55]	0.008
Positive	64	27 (42.2)			
**Mother’s hand contamination (*N*= 200)**					
Negative	163	49 (30.1)	0.715	0.84 [0.36-1.93]	0.684
Positive	37	10 (27.0)			
**Maternal fever during pregnancy (*N*= 200)**					
No	105	23 (21.9%)			
Yes	95	36 (37.9%)	0.013	2.17 [1.17-4.05]	0.014
**Outcome (*N*= 200)**					
Discharge	182	52 (28.6)	0.363	1.63 [0.57-4.57]	0.355
Death	18	7 (38.9)			

#### Phenotypic similarities of MDR-GNB between blood isolates and rectal colonization or bed contamination or mother’s hand contamination

A proportion of 11.7% (7/59), 8.5% (5/59) and 6.8% (4/59) MDR-GNB isolates causing bacteremia had identical bacteria species with MDR-GNB colonizing neonates, contaminating neonates’ beds and contaminating hands of neonates’ mothers, respectively (Table 4).

**Table 4:** AST profiles as a measure of phenotypic similarities between pairs of isolates of MDR-GNB isolated from blood and MDR-GNB isolated from rectal, bed and mothers’ hands swabs

Phenotypic pairs	ID	Isolates	Sources	Comparisons and interpretations of inhibition zones (mm)					
				SXT	TE	CN	CIP	MEM	CT
Blood vs rectal colonization 11.9% (7/59)	068CL	*K. pneumoniae*	Blood	6 (R)	20 (S)	10 (R)	34 (S)	30 (S)	13 (I)
			Rectal	6 (R)	20 (S)	10 (R)	32 (S)	30 (S)	12 (I)
	233CL	*E. aerogenes*	Blood	6 (R)	8 (R)	15 (S)	20 (I)	32 (S)	13 (I)
			Rectal	6 (R)	12 (I)	17 (S)	22 (S)	28 (S)	13 (I)
	275CL	*K. pneumoniae*	Blood	6 (R)	24 (S)	14 (I)	28 (S)	28 (S)	13 (I)
			Rectal	6 (R)	22 (S)	14 (I)	28 (S)	32 (S)	14 (S)
	285CL	*K. pneumoniae*	Blood	6 (R)	6 (R)	6 (R)	17 (I)	30 (S)	15 (S)
			Rectal	6 (R)	6 (R)	8 (R)	20 (I)	30 (S)	15 (S)
	185CL	*Acinetobacter* spp	Blood	22 (S)	18 (S)	20 (S)	22 (S)	32 (S)	13 (I)
			Rectal	6 (R)	6 (R)	10 (R)	10 (R)	23 (S)	14 (S)
	083CL	*K. pneumoniae*	Blood	6 (R)	20 (S)	10 (R)	34 (S)	30 (S)	13 (I)
			Rectal	6 (R)	20 (S)	8 (R)	32 (S)	30 (S)	11 (R)
	282CL	*K. pneumoniae*	Blood	6 (R)	22 (S)	15 (S)	27 (S)	30 (S)	16 (S)
			Rectal	6 (R)	23 (S)	16 (S)	27 (S)	28 (S)	16 (S)
Blood vs bed contamination 8.5% (5/59)	249CL	*K. pneumoniae*	Blood	6 (R)	23 (S)	15 (S)	26 (S)	12 (R)	11 (R)
			Bed	6 (R)	20 (S)	14 (I)	27 (S)	29 (S)	12 (I)
	241CL	*Acinetobacter* spp	Blood	6 (R)	6 (R)	16 (S)	6 (R)	10 (R)	13 (I)
			Bed	6 (R)	6 (R)	10 (R)	13 (R)	8 (R)	14 (S)
	187CL	*Acinetobacter* spp	Blood	6 (R)	8 (R)	15 (S)	25 (S)	27 (S)	13 (I)
			Bed	6 (R)	6 (R)	24 (S)	6 (R)	6 (R)	13 (I)
	242CL	*Acinetobacter* spp	Blood	24 (S)	18 (S)	14 (I)	30 (S)	28 (S)	14 (S)
			Bed	6 (R)	6 (R)	14 (I)	28 (S)	6 (R)	13 (I)
	243CL	*Acinetobacter* spp	Blood	6 (R)	25 (S)	24 (S)	30 (S)	15 (I)	13 (I)
			Bed	6 (R)	6 (R)	15 (S)	27 (S)	6 (R)	15 (S)
Blood vs mother contaminated hand 6.8% (4/59)	068CL	*K. pneumoniae*	Blood	6 (R)	20 (S)	10 (R)	34 (S)	30 (S)	13 (I)
			Hand	6 (R)	11 (R)	6 (R)	22 (S)	26 (S)	15 (S)
	083CL	*K. pneumoniae*	Blood	6 (R)	12 (I)	12 (R)	32 (S)	30 (S)	13 (I)
			Hand	6 (R)	22 (S)	6 (R)	28 (S)	28 (S)	14 (S)
	186CL	*K. pneumoniae*	Blood	6 (R)	18 (S)	10 (R)	28 (S)	28 (S)	14 (S)
			Hand	6 (R)	6 (R)	8 (R)	17 (I)	28 (S)	12 (I)
	294CL	*K. pneumoniae*	Blood	6 (R)	6 (R)	6 (R)	32 (S)	32 (S)	14 (S)
			Hand	6 (R)	22 (S)	6 (R)	15 (R)	28 (S)	16 (S)

Notes: *SXT* trimethoprim-sulfamethoxazole, *TE* tetracycline, *CN* gentamicin, *CIP* ciprofloxacin, *MEM* meropenem and *CT* colistin sulfate, *S* sensitive, *I* intermediate and *R* resistant

## Discussion

Slightly majority (52.5%) of neonates enrolled in this study were males with overall median duration of stay in the respective unit of 7 days however 1 day was the shortest stay and about 23 days was the longest stay. The majority (73%) were enrolled in this study after 48 h of being admitted in the respective unit, suggesting that these neonates developed HCAIs however this was not statistically significant. The majority (87.5%) of neonates were also on antibiotics use during clinical sampling, which may have reduced the sensitivity of culture based diagnostic tests mainly blood culture [31, 32].

In the current study, about one third of neonates had positive culture confirmed bacteremia, despite the fact that a large proportion of neonates were already receiving treatment, which may reduce recover of bacteria from blood culture [31, 32]. Over three quarters of the isolated bacteria from blood cultures were Gram-negative bacteria, of which *K. pneumoniae*, *Acinetobacter* spp. and *E. coli* were frequently isolated. Similar results were reported previously in the same setting, BMC [11] and elsewhere [33].

Significantly large proportion of GNB isolated from blood culture were resistant to 3GCs. In addition, almost 95% of GNB isolated from blood culture and GNB r-3GCs isolated from rectal, cots and mothers’ hands swabs were found to be MDR-GNB. Generally, all MDR-GNB isolated from blood, rectal swabs, bed swabs and hand swabs were more frequently resistant to commonly used antibiotics than uncommon antibiotics. Commonly used antibiotics, such as ampicillin, trimethoprim-sulfamethoxazole, tetracycline, gentamicin, ciprofloxacin, amoxicillin-clavulanate and ceftriaxone, are used as first- and second-line treatment options and as prophylaxis [34]. The MDR-GNB showed low prevalences of resistance against amikacin and meropenem. Regulated use of these antibiotics in Tanzania, as meropenem is reserved for treatment of infections with MDR bacteria and amikacin for treatment of tuberculosis and actinemycetoma, may explain the low bacterial resistance against them [34]. Despite the fact that colistin sulfate is not registered and available for clinical use in Tanzania [34], GNB isolated in our settings exhibited higher percentages of resistance against it. In the same region (Tanzania), one study reported a 66.1% resistance to colistin sulfate among Enterobacteriaceae colonizing hotel employees [35] and another study reported a 95.6% resistance to colistin sulfate among *Campylobacter* spp. isolated from humans [36]. The use of colistin sulfate in veterinary medicine in Tanzania [37], suggests that, veterinary use of antimicrobials may be a key driver of the AMR problems in environment as well as clinical settings as observed in this study.

This current study examined risk factors of bacteremia due to GNB based on pre-admission history, neonatal clinical presentation and potential transmission in the unit; neonatal ICU and/or neonatology unit. Therefore, this study found that, domestic use of tap water only as pre-admission history is protective factor (*p* = 0.040) for bacteremia. Treatment of water for domestic use by sand filtrations at water treatment plant in Mwanza [38], may have been played an effective role of reducing the absolute concentrations of MDR-bacteria and antibiotic resistance genes (ARGs) from contaminated source [39] as reported by Zhang et al, 2016 [40]. Thus, admitted neonates with parents’ domestic use of water from open sources such as dams and lake, should be screened for possibilities of bacteremia due to GNB. Maternal fever during pregnancy is the manifestation of systemic inflammations which may be due to infections such as BSIs, UTIs, infections of the amniotic fluid, or foetal membranes or placenta. Apart from causing maternal complications, these infections may be associated with early onset of neonatal complications such as bacteremia, pneumonia and meningitis [54]. Neonates with clinical presentations of jaundice (*p* = 0.031) and cyanosis (*p* = 0.002) were significantly culture confirmed positive for bacteremia due to GNB. Sepsis induces host production of cytokines (interleukin-1β, tumor necrosis factor-α, nitric oxide and reactive oxygen species), which result in dysregulated systemic inflammatory response associated with multiple organ damage and shock e.g., cardiac dysfunction and hepatocellular injury [41]. Cardiac dysfunction, a cardiopulmonary condition, causes shortage supply of oxygenated haemoglobin (blood) reaching body parts resulting to cyanosis [42]. Further, hepatocellular injury and bacterial products which causes hemolysis e.g., cytolysins, promotes elevation of serum bilirubin leading to jaundice [43]. Therefore, cyanosis and jaundice can be used as accompanying markers in diagnosis of sepsis among neonates in critical care units. Empirical antibiotic therapy, may also be initiated after blood sample collection if neonate presents clinical signs and symptoms of cyanosis and jaundice and whilst awaiting for microbiological culture results. However, third line antibiotic therapy is recommended at this setting as significant higher proportion of GNB isolated from blood culture are resistant to 3GCs and are MDR-GNB, respectively.

Contaminated cots (*p* = 0.008) and multiple invasive devices (*p* = 0.031) suggests potential transmission in the units as they significantly associated with bacteremia. Similar findings were reported elsewhere [44–46]. Invasive devices e.g., intravascular lines required for venous access for administration of medications among critically ill may also provide portal of entry of potential pathogenic bacteria if inserted through contaminated skin [45]. Contaminated inanimate surfaces in the patient’s zone (patient’s immediate surroundings) such as cots increases the risk of healthcare associated infections (HCAIs) mostly among patients with multiple invasive devices [46]. Contaminated hands of HCWs and/or CGs play the major role in cross-transmitting pathogens from contaminated inanimate surfaces to patients resulting to HCAIs [46].

As previously reported [20], rectal colonization with MDR-GNB among neonates in critical care units is high at BMC. This study (43%) and a another study (54.6%) in 2016 [20] found a higher prevalence of neonatal rectal colonization with MDR-GNB than a study (25.4%) conducted in 2013 [19] at BMC. Trends towards increasing prevalence of MDR colonization likely reflect increasing rates of antibiotic resistance. A study conducted from 2013 to 2015 [47] observed high resistance of GNB to 3GCs causing infections at the same setting, Mwanza, Tanzania. Similarly to other studies [19, 48, 49], MDR-*K. pneumoniae* and *Acinetobacter* spp. were the most common GNB r-3GCs and potential Amp-C beta-lactamase producers, respectively, predominantly colonizing neonates in critical care units in our study.

A large proportion (32%) of neonates’ cots were contaminated with MDR-GNB, significantly (*p* = 0.004) associated with rectal colonization of the current neonates occupying the cots. Similar observation, large proportion of inanimate surfaces contamination, was reported previous in similar hospital in Mwanza, Tanzania [21]. The capacity for biofilm formation and multiple mechanisms of resistance to antibiotics, heavy metals and detergents/disinfectants enables long duration survival of contaminating bacteria on inanimate surfaces including cots [46, 50, 51]. Patients’ immediate inanimate surfaces, such as neonatal cots, can be directly contaminated by microorganisms shedded from infected and/or colonized patients as observed in this study that, contamination of neonatal cots is significantly associated with neonate’s rectal colonization. Microorganisms, may also be cross-transmitted to contaminate inanimate surfaces through contaminated hands of healthcare workers (HCWs) and caregivers (CGs) [46]. Overcrowding of neonates, unacceptably small distances between cots and infrequent decontamination of neonates’ cots as observed by this study may lead to increased contamination of neonates’ cots in this settings. Furthermore, other factors including concentration of decontaminant, types of surface contaminating bacteria, contact time with surfaces, and care of cleaning cloth are reported associated with high levels of contamination of inanimate surfaces [39]. CDC recommends regularly decontamination of reusable cleaning cloths and mops [40]. Further, surfaces contaminated with MDR-GNB were found a significant risk factor for bacteremia in critical care units as reported previously [52]. A patient occupying a bed or room after an MDR colonized or infected patient, which was improperly (or not) disinfected, has an increased risk of acquiring infection due to MDR bacteria [52].

Almost one fifth (18.5%) of mothers’ hands were contaminated with GNB r-3GCs in this setting. High proportion (94.6%) of GNB -3GCs, were MDR-GNB. Before touching and breastfeeding their neonates, mothers wash their hands with running tap water and detergents. It is possible that handwashing practices are insufficient or they acquired contamination when touching contaminated surfaces such as beds and/or during other contact with their baby such as diaper changing, as significant number of neonates and beds were colonized and contaminated, respectively. The hands of healthcare workers (HCWs) or caregivers (CGs) after touching contaminated inanimate surfaces such as beds act as vehicles in cross-transmitting MDR bacteria to patients [53]; consequently resulting to patients’ acquisition of infections due to MDR bacteria.

This study observed seven, five and four pairs out of 59 pairs of MDR-GNB isolated from neonatal blood having similar species with MDR-GNB isolated from rectal colonization, cots contamination and mothers’ hands contamination, respectively. This observation may suggests possible cross-transmission of MDR-GNB between these niches [46]. Further, screening of multiple isolates per sample and molecular typing techniques with greater resolution, e.g. multi-locus sequence typing, pulse-field gel electrophoresis (PFGE) or, ideally, whole genome sequencing (WGS) will be important in determining clonal similarities of these isolates.

## Conclusion

Our study found high prevalence of antimicrobial resistant Gram-negative bacteria in sepsis patients in neonatal ICU and neonatology unit. Additionally, high prevalence of MDR-GNB colonizing neonates, contaminating hands of neonates’ mothers and contaminating neonates’ immediate environment, their cots, is extremely concerning. As a result, this study provides evidence for immediate recommendation for: better and frequently (e.g., weekly) decontamination on neonates’ cots; information campaign for mothers on potential cross-transmission of MDR bacteria in causing bacteremia through contaminated hands; and prioritization of 3rd line treatments based on clinical (cyanosis and jaundice) and pre-admission history (domestic use of open water sources) in neonatal intensive care and neonatology units at this setting. Furthermore, a follow-up study is recommended to determine the incidence of bacteremia after proper decontamination protocols are followed up and mothers are educated on infection control practices as recommended.

## Data Availability

The datasets generated and/or analysed during the current study are available in the Microbiology Laboratory Department at Catholic University of Health and Allied Sciences, Bugando, Mwanza-Tanzania.

## Abbreviations

AMC: Amoxicillin-clavulanic acid
AMP: Ampicillin
ARGs: Antibiotic resistant genes
AST: Antibiotic susceptibility testing
BMC: Bugando Medical Centre
CAZ: Ceftazidime
CG: Care giver
CIP: Ciprofloxacin
CLSI: Clinical and Laboratory Standards Institute
CN: Gentamicin
CRO: Ceftriaxone
CT: Colistin sulfate
DDS: Double Disk Synergy
GNB: Gram-negative bacteria
HCAIs: Healthcare associated infections
HCW: Health care worker
ICU: Intensive care unit
IPC: Infection prevention and control
MCA-C: MacConkey Agar supplemented with cefotaxime
MDR: Multidrug resistance
NICU: Neonatal ICU
r-3GCs: resistant to third generation cephalosporins
SBA: Sheep blood agar
TSI: Triple sugar iron
UTI: Urinary tract infection
3GC: Third generation cephalosporin
